# Maternal Immunity in Autism Spectrum Disorders: Questions of Causality, Validity, and Specificity

**DOI:** 10.3390/jcm9082590

**Published:** 2020-08-10

**Authors:** Antonio Ji-Xu, Angela Vincent

**Affiliations:** 1 NHS Foundation Trust, Oxford University Hospitals, Oxford OX3 9DU, UK; antonio.jixu@medsci.ox.ac.uk; 2 Nuffield Department of Clinical Neurosciences and MRC, Weatherall Institute of Molecular Medicine, Oxford OX3 9DU, UK

**Keywords:** maternal immunity, immune activation, animal model, influenza, polyinosinic:polycytidylic acid, lipopolysaccharide, interleukin-6, autism spectrum disorder

## Abstract

Autism spectrum disorders (ASD) are complex neurodevelopmental disorders with unknown heterogeneous aetiologies. Epidemiological studies have found an association between maternal infection and development of ASD in the offspring, and clinical findings reveal a state of immune dysregulation in the pre- and postnatal period of affected subjects. Maternal immune activation (MIA) has been proposed to mediate this association by altering fetal neurodevelopment and leading to autism. Although animal models have supported a causal link between MIA and development of ASD, their validity needs to be explored. Moreover, considering that only a small proportion of affected offspring develop autism, and that MIA has been implicated in related diseases such as schizophrenia, a key unsolved question is how disease specificity and phenotypic outcome are determined. Here, we have integrated preclinical and clinical evidence, including the use of animal models for establishing causality, to explore the role of maternal infections in ASD. A proposed priming/multi-hit model may offer insights into the clinical heterogeneity of ASD, its convergence with related disorders, and therapeutic strategies.

## 1. Introduction

Autism spectrum disorders (ASD) are neurodevelopmental disorders characterised by impairments in social interaction and communication along with restricted/repetitive behaviours [1]. Despite their epidemiological significance, with estimates revealing a rising prevalence of 1 in 68 children, the aetiology of ASD remains unknown. Twin studies have shown a variable but incomplete concordance between monozygotic twins, indicating both genetic and environmental contributions [2,3,4,5]. 

Several lines of evidence implicate the prenatal innate and adaptive immune environment in ASD. Meta-analyses and epidemiological studies have reported an association between maternal infection and incidence of ASD in the offspring [6,7,8], and certain in utero inflammatory markers have been associated with ASD [6]. Animal studies of maternal immune activation (MIA) have characterised a prominent pro-inflammatory phenotype with is associated with impairments in communication and stereotypic behaviours [9,10,11,12,13]. Furthermore, transcriptomic and cytokine profiling studies in individuals with ASD have also revealed a state of immune dysregulation and elevated pro-inflammatory cytokines [14,15,16,17].

However, clinical findings only show correlation and cannot establish a causal link between MIA or maternal antibodies and ASD. Supporting this link are animal models of MIA that serve to elucidate the mechanisms by which fetal development can be altered.

## 2. Search Strategy

The PubMed, Embase, and Web of Science databases were searched on 2 May 2020 using the search strategy: (((prenatal OR mother OR maternal) AND immun*) OR (influenza OR flu) OR (polyinosinic:polycytidylic acid OR polyI:C OR poly I:C OR poly(I:C)) OR (lipopolysaccharide OR LPS) OR (interleukin)) AND (autis* OR ASD OR neurodevelopment*). Reference lists of relevant articles were reviewed to locate additional studies that may have been missed during the search. Search results were restricted to English-language studies only.

## 3. Animal Models of Maternal Immune Activation (MIA)

The main paradigms used to model MIA consist of applying live influenza to pregnant rodents or mimicking infection with immunogenic molecules. Influenza models were pioneered by Fatemi et al. [18] to extend epidemiological findings associating maternal infection with autism and schizophrenia. Maternal influenza administration results in deficits in cortical and hippocampal cytoarchitecture and in social interaction related to ASD [18,19,20] (Table 1). Despite displaying the strongest construct validity, this model does not allow dissociating the effects of the pathogen from those of MIA.

Models based on the bacterial mimic lipopolysaccharide (LPS) or the viral mimic polyinosinic–polycytidylic acid (Poly(I:C)) overcome this limitation and afford precise control of the duration of the immune response. Both induction protocols elicit overlapping findings with maternal influenza injection (Table 1), suggesting that the maternal reaction is more critical than the pathogen per se. Malkova et al. found that poly(I:C) injection during pregnancy resulted in offspring exhibiting the core symptoms of ASD: decreases in sociability and communication and increases in repetitive/stereotypic behaviours [21]. These findings were reproduced by subsequent studies and extended to include other ASD-related symptoms, establishing construct and face validity [10,11,22]. The range of animal models of MIA continues to expand, with more recent models using exposures to Staphylococcal enterotoxin [23], toll-like receptor 7 agonists [24], and allergens [25].

Although these studies support a causal link between MIA and autism, LPS and poly(I:C) injection differ from a live influenza infection, which is acquired through a respiratory route and induces both innate and adaptive arms of immunity. Moreover, maternal infection might have confounding effects on postpartum behaviour, which can influence offspring phenotype [26]. Litter effects, arising from shared genetic factors and maternal environment, may also result in inflated effect sizes. These methodological issues are often neglected and could be overcome by postnatal cross-fostering designs and inclusion of offspring from each litter in investigations, respectively.

It is also unclear to what extent these behavioural findings are representative of ASD symptoms. Although ultrasonic vocalisations (USVs) have been used as a measure of communication deficits [27,28], their intentional communicative nature is unknown, considering the importance of olfactory communication in rodents. Similarly, decreased sniffing behaviour is typically explained in terms of sociability but could be equally ascribed to elevated anxiety or olfactory dysfunction, which should be independently investigated.

Another limitation is that MIA models are restricted by specificity and are unlikely to apply solely to ASD. Most studies have evaluated behaviours that are more relevant to schizophrenia than to autism, such as prepulse inhibition (PPI) and latent inhibition (LI). Behavioural testing is normally conducted during adulthood, which relates more closely to the onset of schizophrenia. Further specificity might be achieved by characterising disease-differentiating features (e.g., antipsychotic response in schizophrenia, stereotyped motor behaviours and macrocephaly in autism); such dissociation might be unfeasible since both diseases share overlapping genetic, neuropathological, and symptomatic traits and could lie across a neurodevelopmental spectrum [29,30].

Future studies could incorporate assays investigating the “theory of mind” deficit in ASD. Mice exhibit greater pain sensitivity after observing cagemates undergo painful stimuli, indicating that some elements of empathy may be testable in mice. Changes in the reward value of social interactions could also be assessed with operant chambers requiring lever presses to access partners. Histological and electrophysiological recordings in the first days of postnatal life are scarce and may inform how neurobiology relates to disease progression. Investigations could also expand to peripheral systems disrupted in ASD patients, such as emerging alterations in gut microbiota [31,32].

Another promising avenue are primate models, due to their complex social structure and their neuroanatomical homology. Patterson and colleagues found that maternal poly(I:C) injection in rhesus macaques led to abnormal social approach, affiliative vocalisations, and repetitive behaviours, validating the MIA primate model [33] (Table 2). Future investigations could analyse ASD deficits that are difficult to explore in rodents, such as joint attention and multisensory temporal integration. Similarly, neuroimaging may be employed to probe the validity of MIA at the level of neural circuitry.

## 4. MIA and Disease Specificity

Epidemiological studies have shown that MIA is a risk factor shared with related disorders including schizophrenia and epilepsy [34]. Although the incidence of infection during pregnancy is estimated at 64%, only around 1% of children develop ASD [8]. This raises a key unsolved question in the MIA field regarding phenotypic outcome: in the presence of prenatal immune challenge, what determines whether the offspring develop ASD, a related disease, or none?

Two hypotheses have been put forward to explain the relationship between MIA and ASD [34,35,36]: (1) MIA constitutes a disease-specific risk factor that depends on inherent characteristics of the immune challenge, or (2) MIA is a general vulnerability factor that predisposes to specific diseases depending on individual genetic, epigenetic, and environmental influences. Below, these two possibilities are discussed.

## 5. Disease Specificity Arising from Immune Challenge

The idea that the properties of the immune challenge (nature, intensity, and timing) can bias progression towards a specific disease is compelling, as different immunogens administered at critical developmental windows could lead to pathogenic cascades manifesting as distinct phenotypes.

Epidemiological evidence indicates that the nature of the immune challenge is less important than the maternal response, with ASD being associated with both bacterial and viral infections [7,8]. Interestingly, a Danish birth cohort study found an association between ASD and maternal viral infection in the first trimester and bacterial infection in the second [8], suggesting that the nature of the pathogen may be important. Major limitations were the exclusion of infectious episodes not requiring hospitalisation and the lack of control for multiple comparisons. In a larger study, Lee et al. observed that the association between maternal infection and ASD was independent of both infectious agent and trimester [7].

The relative importance of the maternal response over the pathogen is also highlighted by animal models, which exhibit similar deficits regardless of the immunogen (Table 1). Nevertheless, there might be subtle differences in the downstream effects of influenza, LPS, and poly(I:C), as they lead to opposite effects in dopaminergic systems [26] and white matter volume [20,37]. These could be related to their respective downstream signalling cascades: LPS acts via Toll-like receptor 4 (TLR4) pathways, including the myeloid differentiation primary response 88 (Myd88) and the TIR-domain-containing adapter-inducing interferon-β (TRIF) dependent pathways. This leads to strong induction of tumour necrosis factor alpha (TNFα). On the other hand, poly(I:C) acts via TLR3 TRIF-dependent pathways mainly inducing type I interferons [38]. Both TNFα and type I and II interferons are increased in the maternal plasma and the blood of individuals with ASD [39,40] and are involved in microglial dysfunction and neurogenesis modulation [14,41,42].

The intensity of the immunogen could also be relevant since different doses may trigger immune responses of varying severity, with disease only occurring when a specific MIA threshold is surpassed. Indeed, rodent models using higher doses of immunogen typically induce more striking behavioural deficits and cytokine alterations in the fetal brain (Table 1; Table 3), and several studies point to a dose-dependent effect of poly(I:C) on behavioural impairments. However, a direct relation between dose and disease development may be over-simplistic, as it ignores maternal variability to immune challenge arising from different gene-environment influences. Studies have revealed that different maternal responses can determine whether offspring develop abnormal behaviour [43].

Timing of prenatal exposure is crucial as immune activation at different critical windows could impact disease-specific neurodevelopmental processes. Although several epidemiological studies support an association between prenatal stressors during mid- and late gestational periods and ASD, others have found associations with early gestation or no association [2,3,7,8]. These discrepancies could be attributed to the lack of substratification in clinical designs, since patient subpopulations may be especially vulnerable to infection due to genetic or environmental factors, compared to others with different aetiologies. In the future, substratification according to infection vulnerability may be conducted to test this hypothesis.

The poly(I:C) model is useful to study timing since it induces a controlled immune reaction lasting 24–48 h [32,44]. Poly(I:C) administration at embryonic day (E) 9 or 12.5 (equivalent to middle-to-late first trimester in humans) typically results in schizophrenia-like behaviour in offspring mice^26^ (Table 1). By contrast, poly(I:C) at E16 (middle second trimester) increases seizure susceptibility while impairing sociability, reminiscent of autism symptoms [45]. Neuroanatomically, mice exposed to early MIA display ventriculomegaly characteristic of schizophrenia, and mice exposed late exhibit decreased cerebrospinal fluid (CSF) volume, perhaps relating to autism. Thus, MIA effects can be dissociated according to the early or late timing of the challenge [46], which may bias development along a spectrum towards schizophrenia or autism-like features, respectively. However, more precise control of the immune reaction is required. This could be achieved with models of reversible interleukin-6 (IL-6) overexpression with tetracycline-controlled transcriptional activation systems, where tetracyclines reversibly switch transcription on or off with spatiotemporal specificity.

It would be informative to interrogate to what extent the neural systems affected at specific stages account for symptom clusters. For example, the hippocampus and corpus callosum develop around E16 in mice. However, although the Black and Tan Brachyury (BTBR) mouse exhibiting agenesis of the corpus callosum is a widely used model of autism [21], the hippocampus is not typically involved in ASD. Collectively, many findings seem to indicate that the characteristics of the immune challenge can result in partly dissociable neuroanatomical and behavioural phenotypes, accounting to a certain extent for disease specificity. Future studies will need to discern whether different immunogens, doses, and administration windows truly result in distinct outcomes within a single study. It will be crucial to use a wider range of measures for ASD and schizophrenia, to avoid falling into a confirmation bias in which results are predetermined by assays capturing specific disease phenotypes. Despite the emergence of neuroinformatic approaches [47], extrapolation of developmental timing from rodents to humans remains spurious, and primate models could help bridge the gap due to their comparably similar development.

**Table 1:** Rodent models of maternal immune activation (MIA).

Reference	Treatment and Species	Timing of Challenge	Behavioural Findings	Neuropathological Findings
Fatemi et al. (1999) [18]	Influenza (IN) Mouse	E9	ND	↓ Reelin expression in cortical layer I and hippocampus ↓ Ventricular size ↑ Brain size Defective corticogenesis, pyramidal cell atrophy
Shi et al. (2003) [19]	Influenza (IN) Mouse	E9.5	↓ PPI ↓ Exploratory behaviour ↓ Social interaction	ND
Shi et al. (2009) [27]	Influenza (IN) Poly(I:C) (20 mg/kg, IP) Mouse	E9.5 (influenza) E12.5 (Poly(I:C))	ND	↓ Purkinje cells in lobule VII of cerebellum Delayed migration of granule cells
Kirsten et al. (2012) [48]	LPS (100 μg/kg, IP) Rat	E9.5	↓ Play behaviour ↓ Social interaction ↓ USVs ↓ Exploratory behaviour ↑ Repetitive behaviour No change in anxiety-like behaviour	ND
Xuan et al. (2014) [49]	LPS (75 μg/kg, IP) Poly(I:C) (20 mg/kg, IP) Mouse	E11.5, 12 (LPS) E12.5 (Poly(I:C))	↓ Locomotor activity * ↓ Social approach * ↑ Repetitive behaviour *	ND
Sharova et al. (2014) [50]	LPS (45 μg/kg, IP) Mouse	E11.5	ND	↓ Neuron number in forebrain area ↑ Neuron number in nasal and olfactory bulb Altered gonadotropin-releasing hormone neuron migration
Foley et al. (2015) [51]	LPS (50 μg/kg, SC) Rat	E15, 16	↑ Acoustic startle * No change in PPI *	ND
Wischhof et al. (2015) [52]	LPS (100 μg/kg, IP) Rat	E15, 16	↓ PPI * ↓ Recognition memory	↓ Myelination in cortex and limbic regions ↓ PARV-expressing interneurons in medial prefrontal cortex, entorhinal cortex, and hippocampus
Batinić et al. (2016) [53]	LPS (100 μg/kg, IP) Rat	E15, 16	↓ Locomotion * ↓ Response to amphetamines * ↓ Spatial learning and memory *	ND
Fernández de Cossío et al. (2017) [28]	LPS (100 μg/kg, IP) Mouse	E15	↓ USV duration ↓ Social behaviour ↑ Stereotypical behaviour ↑ Repetitive behaviour *	Increased spine density in granule cells * No effect on pyramidal cells
Kirsten et al. (2017) [48]	LPS (100 μg/kg, IP) Rats	E9.5	↑ Repetitive behaviour	Reduced dopaminergic activity in the hypothalamus
Wu et al. (2018) [54]	LPS (75 μg/kg, IP) Mouse	E14.5	↑ Anxiety-like behaviour ↓ Social interaction ↑ Depression-like behaviour	Aberrant cytoarchitecture and lamination in neocortex ↓ Intermediate progenitor cells and astrocytes in neocortex
Meyer et al. (2006, 2008) [26,55] Li et al. (2009) [46]	Poly(I:C) (5 mg/kg, IV) Mouse	E9, 17	↓ Exploratory behaviour * ↓ Reversal learning ↓ PPI * ↑ Repetitive behaviour * ↑ Locomotor activity	↓ Hippocampal reelin expression ↓ Ventricular volume * ↑ Dopamine-relater marker expression Altered glutamate-related marker expression
Abazyan et al. (2010) [56] Lipina et al. (2013) [57]	Poly(I:C) (5 mg/kg, IP) Mouse	E9	↓ PPI ↓ LI ↓ Exploratory behaviour Hyperactivity	ND
Hsiao et al. (2012) [44]	Poly(I:C) (20 mg/kg, IP) IL-6 (5 μg, IP) Mouse	E12.5	↓ PPI ↓ LI ↓ Exploratory behaviour	ND
Malkova et al. (2012) [21]	Poly(I:C) (5 mg/kg, IP) Mouse	E10.5, 12.5, 14.5	↓ USVs ↓ Social interaction ↓ Female-induced scent marking ↑ Repetitive behaviour	ND
Ehninger et al. (2012) [58]	Poly(I:C) (20 mg/kg, IP) Mouse	E10.5, 12.5, 14.5	No change in social approach, exploratory behaviour, or olfactory function	ND
Hsiao et al. (2013) [32] Schwartzer et al. (2013) [25] Wu et al. (2015) [29]	Poly(I:C) (20 mg/kg, IP) Mouse	E12.5	↓ PPI ↓ USVs ↓ LI ↓ Social approach ↑ Repetitive behaviour ↑ Anxiety-like behaviour ↑ Depressive-like behaviour	↓ Neuronal proliferation, maturation and survival in dentate gyrus ↓ VEGFA-VEGFR2 hippocampal expression Impaired hippocampal long-term potentiation
Giovanoli et al. (2013) [59]	Poly(I:C) (1 mg/kg, IP) Mouse	E9	↓ LI No change in PPI or anxiety-like behaviour	↑ Reelin expression No change in microglia activation or GABAergic interneurons in CA regions
Missault et al. (2014) [43]	Poly(I:C) (4 mg/kg, IP, IV) Rat	E15	↓ PPI ↑ Depressive-like behaviour No change in locomotor activity	No change in microglial number
Giovanoli et al. (2016) [60]	Poly(I:C) (5 mg/kg, IV) Mouse	E9	ND	Presynaptic hippocampal deficits No change in microglia or astrocyte density
Pendyala et al. (2017) [12]	Poly(I:C) (20 mg/kg, IP) Mouse	E12.5	↓ USVs ↑ Repetitive behaviour No change in motor coordination	Proinflammatory state in cerebellum Reduction in cerebellar synaptic organising proteins
Li et al. (2018) [61]	Poly(I:C) (10 mg/kg, IV) Rat	E9	↑ Locomotion ↓ PPI ↓ LI	Increased prefrontal cortex and hippocampus activity Increased activation of microglia
Murray et al. (2019) [9]	Poly(I:C) (10 mg/kg, IP) Rat	E15	ND	Increased activation of microglia in hippocampus *
Lins et al. (2019) [62]	Poly(I:C) (20 mg/kg, IP) Rat	E15	↓ Social interaction * No change in startle or PPI	ND
Amodeo et al. (2019) [10]	Poly(I:C) (20 mg/kg, IP) Mouse	E12.5	↓ Social behaviour ↓Reversal learning	Dysregulation of potassium ion channel activity in frontal cortex
Carlezon et al. (2019) [22]	Poly(I:C) (20 mg/kg, IP) LPS (10 mg/kg, SC, postnatal day 9) Mouse	E12.5	↓ USVs ↓ Social behaviour * ↑ Anxiety-like behaviour *	Pro-inflammatory state in prefrontal cortex, amygdala, hippocampus and thalamus *
Haida et al. (2019) [11]	Poly(I:C) (20 mg/kg, IP) Mouse	E12.5	↓ Social behaviour * ↓Motor coordination *	Reduced number of Purkinje cells in cerebellum * Reduced number of neurons in motor cortex*
Samuelsson et al. (2006) [63]	IL-6 (9 μg/kg, IP) Rat	E10, 12, 16 or E16, 18, 20	↓ Spatial learning	↓ Neuron number in CA regions ↑ Astrogliosis ↑ Apoptosis ↑ GFAP expression
Smith et al. (2007) [64]	IL-6 (5 μg, IP) Poly(I:C) (20 mg/kg, IP) Mouse	E12.5	↓ PPI ↓ LI ↓ Exploratory behaviour ↓ Social interaction	ND

IN, intranasal; E, embryonic day; ND, not determined; PPI, prepulse inhibition; LPS, lipopolysaccharide; IP, intraperitoneal; IV, intravenous; USV, ultrasonic vocalisations; LI, latent inhibition; poly(I:C), polyinosinic:polycytidylic acid; IL-6, interleukin-6; CA, cornu Ammonis. * Effects were sex-, age-, or genotype-dependent.

**Table 2:** Primate models of MIA.

Reference	Treatment and Species	Timing of Challenge	Behavioural Findings	Neuropathological Findings
Short et al. (2010) [20]	Influenza (IN) Rhesus macaque	Early 3rd trimester	Altered mother-infant interaction	↓ Total and cortical grey matter
Willette et al. (2011) [37]	LPS (2 or 4 ng/kg, IV) Rhesus macaque	Early 3rd trimester	↓ PPI ↑ Emotionality No change in social interaction	↓ Medial temporal lobe grey matter ↑ Total white matter volume
Bauman et al. (2014) [33]	Modified poly(I:C) (0.25 mg/kg, IV) Rhesus macaque	Late 1st and 2nd trimester	↓ Affiliative vocalisations ↓ Social approach ↑ Motor stereotypy Abnormal social behaviour Abnormal attachment to mother	ND
Machado et al. (2015) [65]	Modified poly(I:C) (0.25 mg/kg, IV) Rhesus macaque	Late 1st trimester	↓ Social attention ↓ Visual fixation	ND
Weir et al. (2015) [66]	Modified poly(I:C) (0.25, 0.5, or 1 mg/kg, IV) Rhesus macaque	Late 1st trimester	ND	↓ Apical dendrite size in prefrontal cortex ↑ Number of oblique dendrites
Rose et al. (2017) [67]	Modified poly(I:C) (0.25 mg/kg, IV)	Late 1st or 2nd trimester	↑ Stereotyped behaviours	ND
Bauman et al. (2019) [68]	Modified poly(I:C) (0.25 mg/kg, IV)	Late 1st or 2nd trimester	ND	↑ Striatal dopamine in late adolescence

IN, intranasal; LPS, lipopolysaccharide; IV, intravenous; PPI, prepulse inhibition; ND, not determined; poly(I:C), polyinosinic:polycytidylic acid.

**Table 3:** MIA-induced cytokine increases in the fetal brain of animal models.

Reference	Treatment and Species	Timing of Challenge	Immune Findings
Meyer et al. (2006, 2008) [26,55]	Poly(I:C) (5 mg/kg, IV) Mouse	E9, 17	↑ TNFα, IL-1α, IL-6, IL-10
Abazyan et al. (2010) [56]	Poly(I:C) (5 mg/kg, IP) Mouse	E9	↑ IL-1α, IL-4, IL-5
Arrode-Brusés et al. (2012) [69]	Poly(I:C) (20 mg/kg, IP) Mouse	E16	↑ TNFα, IL-1α, IL-7, IL-13, MCP-1, MIP-1α
Lipina et al. (2013) [57]	Poly(I:C) (2.5 or 5 mg/kg, IV) Mouse	E9	↑ IL-6
Wu et al. (2015) [29]	Poly(I:C) (20 mg/kg, IP) Mouse	E12.5	↑ IL-6
Giovanoli et al. (2016) [60]	Poly(I:C) (5 mg/kg, IV) Mouse	E9	↑ IL-1
Pendyala et al. (2017) [12]	Poly(I:C) (20 mg/kg, IP) Mouse	E12.5	↑ IL-2, IL-3, IL-6, TNFRI, TNF-α, FasL
Rose et al. (2017) [67]	Modified poly(I:C) (0.25 mg/kg, IV) Rhesus macaque	1st or 2nd trimester	↑ IL-1α, IL-4, IL-13

Poly(I:C), polyinosinic:polycytidylic acid; IV, intravenous; E, embryonic day; IL, interleukin; TNFα, tumour necrosis factor alpha; MCP-1, monocyte chemoattract protein-1; MIP-1α, macrophage inflammatory protein-1-alpha; TNFRI, tumour necrosis factor 1; FasL, Fas-ligand.

## 6. Disease Specificity Arising from Genetic and Environmental Factors

Another possibility is that MIA only reveals its neuropathological impact in subjects with genetic predisposition. Several cohorts have highlighted that ASD may have a significant genetic component, with heritability estimates ranging from 50% using a Cox model [70] to 83–97% using liability-threshold models [2,3]. A meta-analysis of twin studies calculated the heritability estimate at 64–91% [4].

Transcriptomic analyses have found that ASD is correlated with gene expression modules corresponding to immune genes and M2-activated microglia [17], suggesting that genetic factors may modulate the fetal response to MIA. Mazina et al. found that ASD individuals with copy number variations and history of maternal infection showed more severe impairments in communication and repetitive behaviours, implying an interaction between genetic susceptibility and prenatal immune challenge [71]. However, this does not necessarily indicate interaction as their effects could be independent and additive rather than synergistic. This could be investigated by exploring whether maternal infection results in ASD solely in subjects with a family history of neurodevelopmental disorders. A meta-analysis of common genetic variants in ASD failed to accurately identify variants that contribute to the aetiology of ASD, suggesting that either common variants do not play a major role in the genetic contribution or that larger population studies may be needed to achieve sufficient statistical power [51].

Multiple multi-trait models have investigated gene-MIA interactions. Ehninger et al. found that MIA resulted in disrupted social behaviour in mice offspring only in the presence of ASD-associated tuberous sclerosis complex 2 (TSC2) mutations [58]. Similarly, expression of mutant human disrupted-in-schizophrenia 1 (mhDisc1) or disrupted in schizophrenia 1 (Disc1) L100P mutations, linked to autism and schizophrenia, revealed synergistic effects with low-dose MIA on social and cognitive deficits, reduced amygdala volume, and elevated IL-6 levels in mice [32]. These findings support the notion that gene-MIA interactions could account for incomplete penetrance in ASD.

However, these genetic models are confounded by the effects of sex and background strain, which can affect the expression of autism-like phenotypes [25,72]. Although most studies only test male offspring, comparison of sex-specific effects may provide insights into the 4:1 male-to-female ratio in ASD. Broader issues concern the validity of using single gene manipulations, since the genetic architecture of ASD in humans is complex and heterogeneous [73], and most genetic risk in ASD arises from common variation, with rare variants contributing only around 3% [74].

Technologies such as the CRISPR/Cas (clustered regularly interspaced short palindromic repeats) system allow targeting multiple genes and could be used to investigate the additive effects of gene variants and their interaction with MIA. However, using transgenic models of common variants of small effect could compromise construct validity. Two categories of genes that could be further examined in combination with MIA are susceptibility factors that do not recapitulate all core symptoms of autism when manipulated alone ((FMRP translational regulator 1 (FMR1), methyl CpG binding protein 2 (MECP2)) and immune-related genes associated with ASD (human leukocyte antigen (HLA), phosphatase and tensin homolog (PTEN)).

On the other hand, the possibility that MIA may require exposure to an additional environmental insult to trigger full-blown pathology remains underexplored. Animal models combining MIA with postnatal stress have found synergistic effects on PPI deficits and microglia activation, supporting this “two-hit” model [59]. Nonetheless, considering the early onset of ASD, secondary environmental insults during pre- or perinatal life may be more relevant, and the examination of MIA convergence with such insults may help develop preventive strategies.

**Figure 1:**
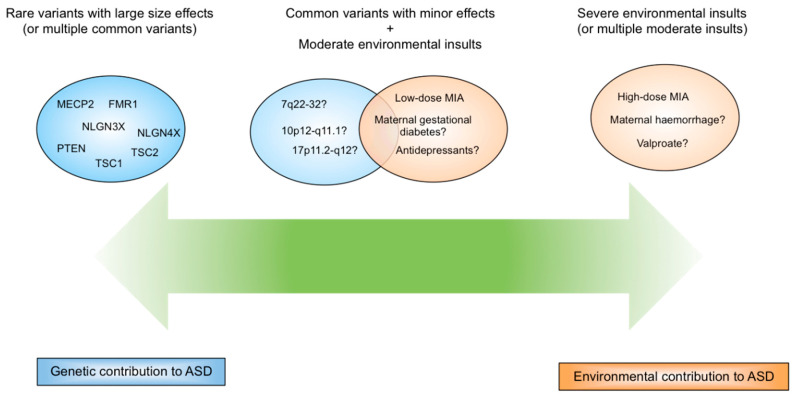
A spectrum of genetic versus environmental contribution in the aetiology of autism spectrum disorder (ASD).

In terms of genetic versus environmental contribution, one can propose a framework in which either extreme genetic predisposition or environmental insults can cause ASD, with a proportion of ASD cases resulting from gene-environment interactions in the middle of this spectrum (Figure 1).

**Figure 2:**
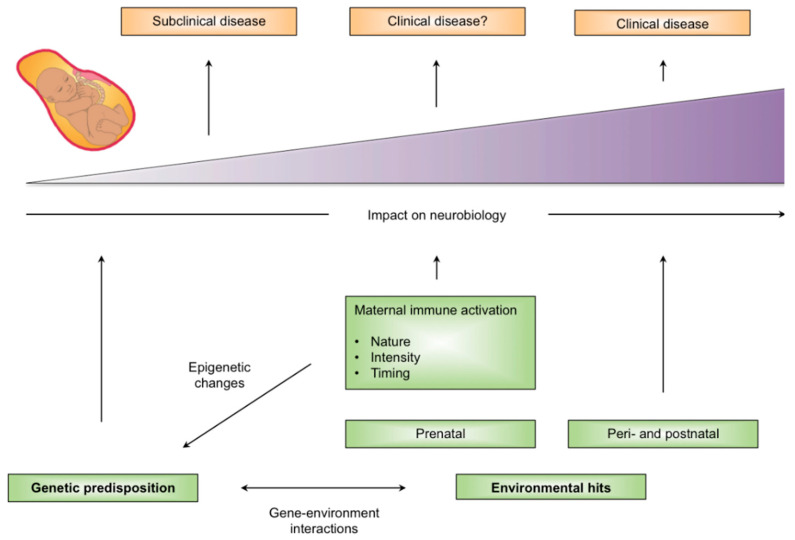
A priming/multi-hit model for MIA and disease specificity.

The hypotheses of disease specificity arising from MIA properties or gene-environment interactions are not mutually exclusive. It is possible to integrate these factors into a single priming/multi-hit model, in which MIA serves as a developmental primer that increases vulnerability towards disease-specific pathways depending on its nature, intensity, and timing. Another layer of phenotypic specificity is provided in the form of genetic, epigenetic, and environmental factors that are required for the progression from vulnerability to full-blown disease (Figure 2). Two meta-analysis of genome-wide association studies have identified novel loci that are shared between ASD and schizophrenia, raising the possibility of a shared pathway for disruption of neurodevelopment in these conditions [75,76].

## 7. Mechanistic Insights

How does MIA result in autism-like behaviours in the offspring? One hypothesis is that an MIA-induced elevation in proinflammatory cytokines alters fetal neurodevelopment [77]. IL-6 has been identified as a key mediator, since maternal injection of poly(I:C) and IL-6 lead to the same behavioural deficits, which are prevented by co-injection of an anti-IL-6 antibody or IL-6 knock-out (KO) [63,64].

**Figure 3:**
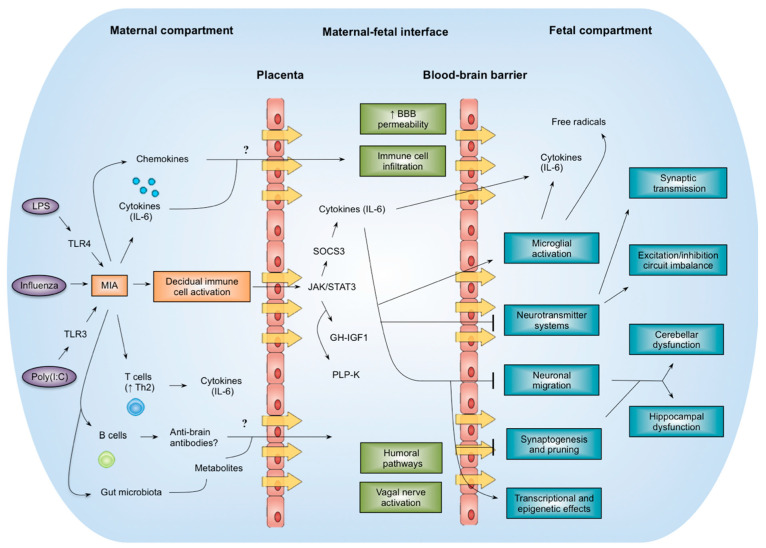
Sites of action and mechanisms of MIA.

Cytokines could exert their effects at three levels: the maternal circulation, the maternal-fetal interface, and the fetal brain (Figure 3). Injection of poly(I:C) into pregnant mice leads to increases in IL-6 in the placenta, derived from uterine natural killer cells and decidual macrophages, and activate the Janus kinase (JAK)-signal transducer and activator of transcription (STAT) pathway in the spongiotrophoblast layer expressing glycoprotein 130 (gp130) and IL-6 receptors [32,44]. Changes in STAT transcription factors increase expression of acute phase genes (suppressor of cytokine signalling 3 (SOCS3), tissue inhibitor of metalloproteinases 1 (TIMP1)) and disrupt the growth hormone (GH)–insulin-like growth factor (IGF)-I axis [32,44]. This constitutes an indirect mechanism by which MIA could alter fetal development through disrupting trophoblast function, nutrient partitioning, and maternal-fetal tolerance.

A more direct mechanism could involve IL-6 crossing the placenta to enter the fetal circulation. However, the ability of cytokines to cross the placenta is disputed. While some studies have shown that radiolabelled IL-6 crosses the rat placenta and that IL-6 is transported across the human placenta ex vivo [78], others have found negligible transport [79]. These discrepancies could be ascribed to differences in measurement timing, as it is unknown how placental permeability to cytokines changes across gestation. Despite being hemochorial, rodent placentas show significant differences in interdigitation with human placentas [80]. Further insights into transplacental transport could be acquired by measuring cytokine concentrations in maternal and fetal compartments of the placenta in women undergoing caesarean section, an approach used to measure glucose transfer [81].

Elevated cytokine levels are found in the fetal brain (Table 3), possibly arising from local expression or from cytokines traversing the blood–brain barrier (BBB) via circumventricular organs or active transport [63]. However, the timing of BBB development and cytokine transporter expression in humans remains to be investigated. It will be equally important to delimit the site of action of IL-6, and this could be accomplished by investigating the effects of MIA in tissue-specific Cre-mediated IL-6R KO models.

Another unsolved question is how an acute immune challenge results in permanent behavioural deficits. MIA could lead to chronic neuronal injury underpinning later pathophysiology [55], or MIA-induced epigenetic alterations could affect the expression of inflammatory mediators in postnatal life [44], resulting in chronic elevations in proinflammatory cytokines responsible for behavioural deficits. This intriguing possibility may be tested through combining in vivo microdialysis with the inhibition of inflammatory mediators in specific brain regions.

Finally, the evidence that maternal antibodies to membrane proteins such as contactin-associated protein-like 2 (CASPR2) and (*N*-methyl-d-aspartate receptor) NMDAR can influence the behaviour of mice in maternal-to-fetal transfer models [82], suggests that MIAs may, by stimulating a maternal immune response, lead to potentially harmful autoantibodies. Alternatively, the infections and the associated cytokine responses could provide an inflammatory milieu that increases the transfer of immunoglobulin G (IgG) antibodies or their pathogenic effects. There are many possibilities that remain unexplored.

## 8. Conclusions

The role of MIA in the development of ASD and related disorders is highlighted by animal models linking prenatal infection and ASD-relevant deficits and clinical studies providing correlative evidence. However, key questions about the validity, mechanism, and the source of disease specificity in this hypothesis remain unsolved. A priming/multi-hit model, in which MIA acts as a primer requiring a substantial genetic contribution and further environmental factors to trigger the clinical manifestation of ASD, could be used in future studies to develop increasingly valid multi-trait animal models. Applying this framework to answer the MIA disease specificity question may offer insight into the clinical heterogeneity of ASD and its convergence with related diseases such as schizophrenia, with the ultimate goal of guiding diagnostic classification and therapeutic strategies in neurodevelopmental psychiatric disorders.
